# Impaired Attribution of Emotion to Facial Expressions in Anxiety and Major Depression

**DOI:** 10.1371/journal.pone.0015058

**Published:** 2010-12-01

**Authors:** Liliana R. Demenescu, Rudie Kortekaas, Johan A. den Boer, André Aleman

**Affiliations:** 1 Department of Neuroscience, University Medical Center Groningen, BCN Neuroimaging Center, University of Groningen, Groningen, The Netherlands; 2 Department of Psychiatry and Psychotherapy, RWTH Aachen University, Aachen, Germany; 3 Department of Psychiatry, University Medical Center Groningen, Groningen, The Netherlands; The University of Melbourne, Australia

## Abstract

**Background:**

Recognition of others' emotions is an important aspect of interpersonal communication. In major depression, a significant emotion recognition impairment has been reported. It remains unclear whether the ability to recognize emotion from facial expressions is also impaired in anxiety disorders. There is a need to review and integrate the published literature on emotional expression recognition in anxiety disorders and major depression.

**Methodology/Principal Findings:**

A detailed literature search was used to identify studies on explicit emotion recognition in patients with anxiety disorders and major depression compared to healthy participants. Eighteen studies provided sufficient information to be included. The differences on emotion recognition impairment between patients and controls (Cohen's d) with corresponding confidence intervals were computed for each study. Over all studies, adults with anxiety disorders had a significant impairment in emotion recognition (d = −0.35). In children with anxiety disorders no significant impairment of emotion recognition was found (d = −0.03). Major depression was associated with an even larger impairment in recognition of facial expressions of emotion (d = −0.58).

**Conclusions/Significance:**

Results from the current analysis support the hypothesis that adults with anxiety disorders or major depression both have a deficit in recognizing facial expression of emotions, and that this deficit is more pronounced in major depression than in anxiety.

## Introduction

The ability to identify and interpret facial expressions of emotion is essential in human communication and social interaction. Ekman and Friesen [1] concluded that six facial expressions are universal across cultures: happy, angry, sad, anxious, disgusted and surprised and each of them is characterized by a particular facial muscular pattern.

Discrimination of emotion from facial expressions has been the focus of a number of psychological studies over the past decades, and was later complemented by neurobiological findings [2], [3]. The specific way in which an individual processes and interprets emotional information can be a causal factor in the development or maintenance of emotional disturbances. Studies in subjects with emotional disorders, such as major depression and anxiety, aim to understand the relation between emotional processing and psychopathology.

Emotional dysfunctions (e.g., difficulty in understanding emotions, difficulty in changing how one feels) are related to poor social functioning and can be considered as important features of psychopathology [4], [5]. Poor social and interpersonal relations could arise from a deficit in the ability to read signals of interpersonal threat or safety. Psychopathological variables may explain the variance in the accuracy of recognition of facial expression of emotions.

Over the last two decades, research regarding emotional facial expressions in anxiety and major depression has focused on two areas: attentional bias and the ability to recognize emotions, with much more consideration devoted to the former. Several types of cognitive bias have been described in social phobia, generalized anxiety disorder and panic disorder. Biases involve attention, judgment, interpretation, imagery and memory (for a review, see: Clark and McManus [6], Hirsch and Clark [7], Lang and Sarmiento [8]). Anxiety disorders have been associated with a selective attentional bias toward threatening stimuli, whereas in major depression a selective attentional bias has been observed for negative emotional stimuli such as those related to sadness, loss and failure [9], [10].

Cognitive deficits, such as perception and attention as well as motivational deficits have been associated with depression [11], [12]. Anxiety has been associated with cognitive operations that “serve the function of maintaining vigilance” and a “reduction of cognitive resources available for other tasks” (see for review Mathews, 1990 [13]). These deficits may affect the way in which emotional facial expressions are processed.

The aim of the present review was to determine, by conducting a comprehensive meta-analytical synthesis of previous studies, the magnitude of the impairment in facial emotion recognition accuracy associated with anxiety disorders and major depression. In children with anxiety disorders there is evidence suggesting an impairment of emotion recognition, however the findings are inconsistent. Because such an impairment may provide clues about the role of developmental processes in anxiety disorders, we examined the extent to which this impairment is present in children with anxiety. We hypothesized that there would be a small difference for anxiety patients when compared to healthy controls in emotion recognition as they do not have substantial cognitive deficits. In contrast, we expected to find a larger impairment in emotion recognition in depressed patients.

## Methods

### Screening procedures and inclusion criteria

The Web of Science (ISI) and PubMed databases were searched for the period 1980–2009. The search was performed using ”anxiety” and ”depression” combined with ”emotion”, ”facial expressions”, ”recognition”, ”discrimination”, ”labeling” as search terms. Additionally, more specific terms were used such as ”social phobia” (SP), ”generalized anxiety disorder” (GAD), ”panic disorder” (PD), ”posttraumatic stress disorder” (PTSD) and ”major depressive disorder” (MDD).

173 studies on anxiety disorders and 208 studies on depression were identified. Subsequently, title and abstract of the articles were screened for possible inclusion in the analysis. The identified studies were included if they met the following criteria: the diagnosis of major depressive disorder or anxiety disorders was made according to the DSM-III or DSM-IV criteria. Secondly, each study had to deal with a group of adults or children experiencing major depression or anxiety disorders and a control group. Third, behavioral measures of emotional facial expressions discrimination or identification accuracy had to be reported with sufficient statistical information for the computation of the effect size (d-value). This implies that means and standard deviations, t-values or F-values and the relevant means, and exact p-values had to be reported. Lastly, only studies published in English were included.

### Description of the paradigms used in the studies

In this study we included facial expression discrimination paradigms measuring emotion recognition accuracy employing presentation of schematic or photographed facial expressions depicting neutral, emotional or ambiguous expressions. These stimuli were either presented on a computer screen, or in a booklet. The duration of the stimulus presentation was either fixed or unrestrained. Participants had to label each facial expression or they had to judge whether a pair of faces expressed the same emotion are or not. Responses were given either by pressing a key or by selecting one of the cards. The accuracy was one of the measures in the emotion recognition paradigms.

### Data analysis

For each study the effect size (Cohen's d) was calculated for the difference in emotion recognition performance between the patient group and the control group. The d was calculated as the difference between the two group means, patient group minus control group and divided by the pooled standard deviation [14]. When means and standard deviation were not given, d-values were computed from F-values or t-values. The effect size was computed using the program developed by D. Wilson (http://mason.gmu.edu/dwilsonb/ma.html). The direction of the effect size was negative if the performance of the patient group in discrimination of facial expression accuracy was worse than the control group.

After computing the effect size for each study, a meta-analytic method was used (Comprehensive Meta-analysis program, www.meta-analysis.com). The combined effect size was calculated with the corresponding confidence intervals (95%) indicating the magnitude of the effect across all studies. The Z-values and p-values provide an indication of the statistical significance of the association. In addition, the Q-statistic was calculated [15] as an indicator of homogeneity. A significant Q-statistic points to heterogeneity of the effects across studies. As the Q test is reported to be susceptible to the number of studies included in the meta-analysis, the I-squared [16] was also calculated. I-squared is an index of heterogeneity describing the percentage of non-chance inconsistency. I-squared of 25% indicates low, 50% moderate and 75% high heterogeneity [16].

Publication bias was tested using the Duval and Tweedie's trim and fill, and by inspecting the funnel plot. The Duval and Tweedie's trim and fill is a nonparametric method which concerns a simple funnel plot-based method of testing and adjusting for publication bias in meta-analysis, by using the ranks of the absolute values of the observed effect sizes and the signs of those effect sizes around the global effect size [17].

## Results

### Search results

Out of 381 identified studies, twenty-eight studies were potentially eligible for inclusion based on screening of the title and abstract, and the full text version of each of these manuscripts was further evaluated. Six studies were excluded because of insufficient data needed to calculate the effect size [18]–[23]. Two studies [24], [25] were excluded as they examined facial emotion recognition in non-clinical participants (high and low anxiety). One study [26] was excluded because it did not include a patient group. One study was excluded because of significant differences in age between the patient group and the control group [27]. Two studies were excluded because of methodological differences [28], [29]. Ten studies targeting facial expression of emotion discrimination in anxiety disorders and eight studies in major depression met our inclusion criteria. Characteristics of the included studies and data are provided in Table S1 (online supporting information, references [30], [31], [32], [33], [34], [35], [36], [37], [38], [39]) for the studies on anxiety disorders and Table S2 (online supporting information, references [23], [40], [41], [42], [43], [44], [45], [46]) for the studies on major depression.

### Meta-analysis results

The results of meta-analysis of emotion recognition in anxiety disorders and major depression are displayed in Table 1. Results from the meta-analysis suggest that children with anxiety disorders do not have an impairment (p = 0.831, d = −0.03) of recognition of facial expression of emotions (Table 1). The effect size after adjustment for possible publication bias using Duval and Tweedie's trim and fill method remained insignificant (d = 0.08, 95% confidence interval: −0.16 to 0.32).

**Table 1:** Meta-analytic results for facial expression of emotion discrimination in children with anxiety disorders, adults with anxiety disorders and adults with depression.

	k	*d*	95% CI	Z	p*	I^2^	Q	p**
Anxiety - Children	5	−0.03 (0.08)	−0.30, 0.24	−0.21	0.831	0.00	2.99	0.560
Anxiety - Adults	5	−0.35 (−0.51)	−0.61, −0.10	−2.69	0.007	22.30	5.15	0.270
Depression - Adults	8	−0.58 (−0.42)	−0.79, −0.36	−5.17	0.000	33.75	10.57	0.160

*Note:* k - number of studies included in the meta-analysis; d - effect size (estimated effect size after correction of publication bias in parentheses); CI - confidence intervals; p* - indicates the statistical significance of association Z; p** - indicates the significance of Q-statistic; I^2^ – indicator of heterogeneity.

In adults with anxiety disorders the meta-analysis showed a significant impairment (p = 0.007) with a medium magnitude (d = −0.35, 95% confidence interval: −0.61 to −0.10) of facial emotion recognition in anxiety disorders. There was a low variability among the effect sizes (I^2^ = 22.30). Correcting this for publication bias still resulted in a robust estimated effect size of d = −0.51 (95% confidence interval: −0.73 to −0.29). The overall effect size was larger than if we ignored a possible publication bias. More specifically, Duval and Tweedie's trim and fill suggested that there may be two missing studies with a negative effect (impaired emotion recognition in anxiety disorders compared to controls).

The meta-analysis of studies on major depression showed significant impairment of emotion recognition with a medium overall effect size of d = −0.58 (p<0.001). There was a moderate variability among the effect sizes (I^2^ = 33.75, Q = 10.57, p = 0.16). Once corrected for publication bias the relationship between the impairment of emotion recognition and major depression remained robust, although this overall effect size was reduced (d = −0.42, 95% confidence interval: −0.62 to −0.23) relative to that estimated from the original data.

## Discussion

The purpose of this study was to investigate whether and to what extent anxiety disorders and major depression are associated with impaired recognition of emotion in others. The results from the current analysis support the hypothesis that adults with major depression or anxiety disorders have an impaired recognition of facial expression of emotion, as substantiated by the medium effect size. This effect was not observed in children with anxiety disorders.

The present meta-analysis shows that children with anxiety disorders do not have an overall emotion recognition deficit. Some studies [32] found that children with anxiety did have an impairment on this task, whereas others [20], [22], [34], [33] found no impairment. Various reasons may account for the inconsistencies between studies, *e.g.*, sample characteristics, the instruction to subjects, the use of adult or child faces or an emotion specific deficit in the children [31], [18]. None of these possible causes could be investigated in the present meta-analysis. However, our results clearly suggest that there is no gross overall impairment in children.

Furthermore, the present meta-analysis revealed a moderate impairment of facial emotion recognition in adults with anxiety disorders. The underlying mechanism for this impairment is unknown, but attentional biases might be involved. Indeed, an emotion specific impairment has been suggested in association with anxiety disorders. For example, Kessler et al. [37] and Mohlman et al. [36] found that socially anxious patients, as compared to healthy participants, had a tendency to misclassify neutral expressions as angry. On the other hand, a high sensitivity has been found in recognizing negative facial expression [47]. Surcinelli et al. [25] reported that non-clinical participants with a high trait anxiety have a better recognition of fearful faces. A possible explanation might be that anxious subjects have a negative bias, such that they misinterpret neutral expressions as displaying a negative emotion [35], [48], [49]. This impairment may also be triggered by the presence of emotional dysregulation in anxiety disorders [50]. As most studies in the present meta-analysis did not report emotion specific recognition scores, we were not able to systematically investigate possible biases.

With regard to major depression, we also found a moderate overall emotion recognition impairment. Thus, patients with major depression may be compromised in recognizing emotions of other people from facial expressions. This may contribute to social dysfunction, as it has been well established that emotion recognition contributes to proficient social functioning [4], [5], [51]. One explanation for this perceptual impairment might be the presence of cognitive deficits associated with major depression, as suggested by Persad and Polivy [43].

Some studies have reported specific emotion recognition impairment in depression. For example, Leppanen et al. [41] reported that depressed patients have an impairment in recognizing neutral faces and a tendency to interpret happy faces as neutral, and interpreting this as a negative shift in emotion recognition [42], [52], [53]. On the other hand, Persad and Polivy [43] reported no specific impairment of emotion recognition, but rather a generalized, non-specific deficit of emotion recognition. The authors suggested that this deficit might also be because of a “lack of attentiveness to others” and a focus that is more directed to patients' own problems [43]. Thus, taken together it may be argued that deficits in recognition of facial expressions of emotion in major depression may be determined by patients' negative emotional experience as well as by the assessment of their internal mood state. Major depression is characterized by negative cognitions (worthlessness, self-criticism, hopelessness) and consequently their evaluation of external stimuli, including facial expressions, might be more negative than in healthy subjects [5], [44].

Another factor which might contribute to the present result is illness severity. Almost all studies included in the present review, with the exception of Archer et al. [45] -- where the severity of the depressive disorder is not clear -- were on patients with moderate-to-severe or severe depression. Thus, this finding may be limited to only patients with moderate-to-severe or severe depression, whereas patients with remitted depression may show no emotion recognition impairment. Venn et al. [54] suggested that emotion recognition impairment is a “state-dependent effect”. On the other hand Bourke and colleagues [55] in their review, indicated that it is not clear yet if impairment of emotion recognition is state or trait marker of depression severity. Further studies may need to examine this effect. Taken all together we may conclude that severe and moderate depression is associated with moderate emotion recognition impairment.

Emotion recognition has been shown to be related to social functioning [56]. Indeed, neuroimaging studies have reported that to a large extent regions involved in emotional processing are also part of the neural network responsible for social cognitive processes [57]. Keightley et al. [58] reported that prefrontal cortical structures, amygdala and inferior temporal cortex (fusiform gyrus) have a critical role in emotion recognition processes. At the neural level, anxiety disorders are associated with amygdala and insula hyperactivation during perception of threat-related emotions [59]. In major depression abnormal cerebral blood flow (CBF) has been shown in amygdala, anterior cingulate cortex (ACC), ventral striatum, anterior insula and prefrontal cortex [60], [61]. Thus, neuroimaging studies can offer an identification of pathological mechanisms associated with affective disorders.

Factors which might influence our results are the small sample size, differences in stimulus material and task complexity, and duration of illness which may have an impact on the outcome of emotion recognition tasks and cognitive tests. A second limitation concerns the fact that there were differences in the degree of severity of the anxiety disorders, not all of them being clinical patients. Another limitation is that we could not distinguish between individual emotions, because most studies did not report adequately detailed data to permit such comparisons. It would be of interest to investigate whether patients with major depression are selectively more impaired for certain expressions (e.g., happy) than others (e.g., sad), as was suggested by Surguladze et al. [23]. Medication use might have been a confounding variable because manipulation of the serotonin system, which is a common antidepressant treatment, produces specific alterations in the ability to recognize fear [62].

In summary, we reviewed behavioral studies indicating the relevance of facial expression of emotion to anxiety disorders and major depression. The present findings suggest a global deficit in recognition of different types of emotions, which was more pronounced in major depression than in anxiety disorders. These emotion recognition deficits may contribute to compromise social functioning in these disorders.

## Supporting Information

Table S1 Characteristics of included studies on anxiety disorders.
(DOC)

Table S2 Characteristics of included studies on major depressive disorder.
(DOC)
