# Supplementary material for: Impaired Attribution of Emotion to Facial Expressions in Anxiety and Major Depression
Source: PLoS One. 2010 Dec 1;5(12):e15058. doi: 10.1371/journal.pone.0015058 (PMC2995734; doi:10.1371/journal.pone.0015058)
Supplement: Table S1 — Characteristics of included studies on anxiety disorders. (DOC) [file pone.0015058.s001.doc]

Table S1: Characteristics of included studies on anxiety disorders

| **Reference** | **Subjects** | **Mean age (years)** | **Psychopathological measures** | **Anxiety rating score** | **Characteristics of the task** | **Effect size: Cohen’s *d* (95% CI)** |
| --- | --- | --- | --- | --- | --- | --- |
| Melfsen & Florin [30] | 17 social anxiety;  15 controls. | 10.24  10.07  Range: 8-12 | Social Phobia and Anxiety Inventory for Children (SPAI-C) | M (s.d.) = 25.72 (8.21);  M (s.d.) = 5.23 (2.09) | 72 pictures with neutral, positive (joyful) or negative (angry, disgusted, sad) facial expressions, black and white, half of them showing adults and half children (Matsumoto and Ekman, 1988).  Emotion identification – forced choice response. | *0.06*  (-0.43 to 0.55) |
| Simonian et al. [31] | 15 social phobia (SP);  14 controls. | 12.2  11.0  Range: 9-15 | SPAI-C | Score ≥ 18 (social phobia)  Score ≤ 15 (control group) | 36 slices from pictures of facial affect, black and white, consisting of adult faces, displaying six emotions (happiness, anger, sadness, fear, surprise and disgust).  Emotion identification – forced choice response. | *-0.24*  (-0.97 to 0.49) |
| Easter et al. [32] | 15 anxiety disorder (11 met criteria for GAD, 8 with SP, 3 with SAD, 4 had comorbid major depression);  11 controls. | 13.1  12.5 | Kiddie-Schedule for Affective Disorders and Schizophrenia for School Age Children (K-SADS-PL).  Pediatric Anxiety Rating Scale. |  | Child facial expression and adult facial expressions subtests of the DANVA (Diagnostic Analysis of Nonverbal Accuracy), consisting of 24 photographs of either children's or adults’ faces displaying happy, sad, angry and fearful.  Emotion identification – forced choice response. | *-0.48*  (-1.28 to 0.31) |
| Manassis & Young [33] | 14 children with anxiety disorders;  10 healthy control children. | 10.5  10.4  Range: 8-12 | Anxiety Disorders Interview Schedule for DSM-IV: Child and Parent versions (ADIS-C/P). |  | DANVA2: 24 item series of adult facial expressions depicting happiness, sadness, anger and fear.  Emotion identification – forced choice response. | *-0.11*  (-0.68 to 0.47) |
| Allen et al. [34] | 20 children with anxiety disorders (GAD, separation anxiety disorder, specific phobia, PTSD);  19 control children. | 9  8.9  Range: 7-15 | Revised Child Manifest Anxiety Scale (RCMAS);  ADIS-C/P |  | Photographic images of facial emotion expressions depicting: surprise, anger, happiness, fear, disgust, sadness.  Categorize each picture as one of the six target emotions using labeled boxes. | *0.29*  (-0.28 to 0.88) |
| Winton et al. [35] | 13 anxious ( high score on the FNE);  11 controls (low score on the FNE). | 20.6  22.7 | Fear of Negative Evaluation Scale (FNE),  Social Avoidance and Distress scale (SADS), Beck Depression Inventory (BDI) | M(s.d.) = 22.5(3.6) on FNE, M(s.d.) = 8.5(8.2) on SADS, M(s.d.) = 8.0(5.2) on BDI;  M(s.d.) = 5.3(2.5) on FNE, M(s.d.) = 2.3(2.7) on SADS, M(s.d.) = 3.2(4.1) on BDI. | 40 slides of negative facial emotional expressions (anger, sadness, disgust, contempt, and fear) and 40 slides displaying neutral expressions (Matsumoto and Ekman, 1988).  Emotion identification – forced choice response. | *-0.39*  (-1.20 to 0.42) |
| Mohlman et al. [36] | 26 GSAD (4 dysthymic disorder, 2 GAD, 2 panic disorder, 1 MDD, 6 specific phobias);  26 controls. | 21.46  21.08 | FNE, Social Phobia Scale (SPS, Mattick & Clarke, 1998), State Trait Anxiety Inventory (STAI), BDI. | GSAD criteria score above 20 on FNE,  Controls scored below 9 on FNE. | Facial expressions depicted: neutral, happy, sad and angry at different affective intensities (25%, 50% and 100%).  Match cards based on emotion depicted on the card. | *-0.22*  (-0.76 to 0.32) |
| Kessler et al. [37] | 37 panic disorder (PD) outpatients;  43 controls. | 37.8  36.4 | State Trait Anxiety Inventory (STAI), BDI. |  | FEEL (Facially Expressed Emotion Labeling) test, portrait pictures, conditions: anger, sadness, disgust, happiness, fear, surprise (Kessler et al., 2002)  Emotion identification – forced choice response. | *-0.74*  (-1.20 to -0.30) |
| Corcoran et al. [38] | 36 PD;  36 controls.  The patients were recruited from anxiety disorder specialty clinics. | 18.1  34 | Anxiety Disorders Interview Schedule for DSM-IV;  Structured Clinical Interview for DSM-IV;  Yale-Brown Obsessive-Compulsive Scale;  BDI. |  | Black and white photographs depicting anger, disgust, fear and sadness (Ekman & Friesen, 1979).  Emotion identification – forced choice response. | *0.04*  (-0.54 to 0.62) |
| Campbell et al. [39] | N = 12 generalized social phobia (GSP);  N = 28 healthy controls (HC) | 31.9  30.4 | Liebowitz Social Anxiety Scale (LSAS);  State-Trait Anxiety Inventory (STAI);  BDI | GSP: M(s.d.) = 90.6 (26.4) on LSAS, M(s.d.) = 45.8 (11.4) on STAI and M(s.d.) = 15.8 (10.6) on BDI;  HC: M(s.d.) = 11.5 (10.8) on LSAS, M(s.d.) = 22.6 (4.3) on STAI and M(s.d.) = 1.8 (2.5) on BDI | 24 emotional faces selected from Matsumoto and Ekman (1988) set depicting: happiness, disgust and anger.  Emotion labeling – forced choice task. | *-0.19*  (-0.87 to 0.49) |
