# Supplementary material for: Impaired Attribution of Emotion to Facial Expressions in Anxiety and Major Depression
Source: PLoS One. 2010 Dec 1;5(12):e15058. doi: 10.1371/journal.pone.0015058 (PMC2995734; doi:10.1371/journal.pone.0015058)
Supplement: Table S2 — Characteristics of included studies on major depressive disorder. (DOC) [file pone.0015058.s002.doc]

Table S2: Characteristics of included studies on major depressive disorder.

| **Reference** | **Subjects** | **Mean age (years)** | **Psychopathological measure** | | **Depression ratings** | **Characteristics of the task** | **Effect size: Cohen’s *d* (95% CI)** | |
| --- | --- | --- | --- | --- | --- | --- | --- | --- |
| Rubinow & Post [40] | 17 inpatients with affective disorders (7 bipolar I, 5 bipolar II, 5 unipolar),  31 controls. | 39  31 | Bunney-Hamburg Depression Scale (B-HDS)  Research Diagnostic Criteria (Spitzer et al, 1978) | | ≥ 7 on B-HDS in inpatients. Patients had moderate-to-severe depression. | 48 photographs of faces: sad, fearful, happy, angry, disgusted, surprised and interested (Ekman et al., 1973).  Match photographs of facial expression with seven key photographs. | *-1.01*  (-1.64 to -0.38) | |
| Leppänen et al. [41] | 18 depressed patients,  18 controls | 45.1  44.7  Range: 23-59. | BDI;  Positive and Negative Affect Scale. | | M(s.d.) = 36.8 (9.6) on BDI in depressed subjects,  M(s.d.) = 11.1. (8.4) on BDI in controls;  M(s.d.) = 25.2 (7.0) on positive affect and 19.7 (9.2) on negative affect in depression,  M(s.d.) = 31.7 (5.0) on positive and 11.8 (2.6) on negative affect in controls.  Patients had moderate-to-severe depression. | 96 trials. Male and female models with happy, sad and neutral expressions selected from Ekman and Friesen (1976).  Emotion identification – forced choice response. | *-1.00*  (-1.70 to -0.31) | |
| Zuroff & Colussy [42] | 15 depressed inpatients (7 dysthymic disorder, 5 MDD, 3 adjustment disorder with depressed mood),  15 controls | 37 | BDI ,  D-30 scale from the MMPI | | M = 14 on BDI and M = 79.5 on D-30 in depression;  M = 1.8 on BDI and M = 44.8 on D-30 in controls. Patients had moderate-to-severe depression. | 32 black and white prints of adult male and female faces (Izard, 1971). Eight emotions: happiness, anger, surprise, disgust, shame, fear, sadness and interest.  Match photographs of facial expressions with the correct label for the emotion. | *-0.70*  (-1.44 to 0.04) | |
| Persad &Polivy [43] | 16 DCS (depressed college students),  16 NDCS (nondepressed college students),  16 DPP (depressed psychiatric patients). | 26.50  Range: 18-53. | BDI | | M = 16.19 on BDI in DCS,  M = 3.75 on BDI in NDCS.  -diagnosis of MDD on Axis I, according to DSM-III, a cutoff score of 22 or higher on BDI in DPP. | Facial affective booklet, consisting of a set of 14 photographed facial expressions developed by Ekman (1976); expressed emotions: fear, anger, surprise, contempt, happiness, sadness and indifference.  Label facial expression of emotions. | *-0.62*  (-1.36 to 0.13) | |
| Hale [44] | 48 depressed subjects (28 outpatients received antidepressant medication, 15 received benzodiazepine medication),  48 controls. | 38  41  Range: 20-69. | | BDI.  Patients were included if the BDI score was ≥ 17. | M(s.d.) = 27.8 (7.1) on BDI in depression.,  M(s.d.) = 2.7 (2.6) on BDI in control group. | 12 schematic facial expressions of which three expressions were ambiguous/mixed emotions.  Label facial expression. | *-0.52*  (-0.93 to -0.11) |  |
| Archer et al. [45] | 12 depressed inpatients (MDD diagnosis, medication use);  12 controls | 56.19  48.04 | | Hospital diagnosis criteria based on the DSM-III Manual. |  | Facial expression of emotion (Ekman and Friesen, 1976) depicted: happy, sad, frightens, angry, surprised, and disgust. Emotions were presented in pairs, subject had to indicate which of the two faces was expressing the target – word presented on the screen. | *-0.52*  (-1.10 to 0.06) |  |
| Surguladze et al. [23] | 27 depressed inpatients and outpatients (MDD diagnosis, medication use);  29 controls. | 46.9  43 | | BDI ;  Hamilton Depression Rating Scale (HAMD). | M(s.d.) = 33 (9.9) depressed patients and M(s.d.) = 3.1 (3.5) controls on BDI;  M(s.d.) = 16.9 (5.5) depressed patients on HAMD. Patients had chronic, recurrent depressive disorder. | 10 facial expressions from standardized series Young et al., (2002), displaying happy, sad and neutral expressions.  Label facial expression. | *-1.21*  (-2.47 to 0.05) |  |
| Kan et al. [46] | 16 depressed inpatients (MDD diagnosis, medication use);  20 controls | 50.9  59.0 | | Hamilton Depression Rating Scale (HDRS);  Zung self rating depression scale (SDS) | M(s.d.) = 18.3 (8.64) on HDRS and M(s.d.) = 60.0 (15.3) on SDS in depressed patients. | Videotaped facial expression from neutral to emotion to neutral. Six basic emotions: happy, sad, angry, fearful, surprise and disgust were presented. Match one basic emotion with the expression presented on the screen. | *0.15*  (-0.43 to 0.73) |  |
